# Losing the beat: deficits in temporal coordination

**DOI:** 10.1098/rstb.2013.0405

**Published:** 2014-12-19

**Authors:** Caroline Palmer, Pascale Lidji, Isabelle Peretz

**Affiliations:** 1 Department of Psychology, McGill University, Montreal, Quebec, Canada H3A 1B1; 2 Center for Research on Brain, Language and Music, McGill University, Montreal, Quebec, Canada H3G 2A8; 3 Department of Psychology, University of Montreal, Montreal, Quebec, Canada H2V 2S9

**Keywords:** entrainment, temporal adaptation, beat deafness, synchronization, endogenous frequency, error correction

## Abstract

Tapping or clapping to an auditory beat, an easy task for most individuals, reveals precise temporal synchronization with auditory patterns such as music, even in the presence of temporal fluctuations. Most models of beat-tracking rely on the theoretical concept of pulse: a perceived regular beat generated by an internal oscillation that forms the foundation of entrainment abilities. Although tapping to the beat is a natural sensorimotor activity for most individuals, not everyone can track an auditory beat. Recently, the case of Mathieu was documented (Phillips-Silver *et al.* 2011 *Neuropsychologia* **49**, 961–969. (doi:10.1016/j.neuropsychologia.2011.02.002)). Mathieu presented himself as having difficulty following a beat and exhibited synchronization failures. We examined beat-tracking in normal control participants, Mathieu, and a second beat-deaf individual, who tapped with an auditory metronome in which unpredictable perturbations were introduced to disrupt entrainment. Both beat-deaf cases exhibited failures in error correction in response to the perturbation task while exhibiting normal spontaneous motor tempi (in the absence of an auditory stimulus), supporting a deficit specific to perception–action coupling. A damped harmonic oscillator model was applied to the temporal adaptation responses; the model's parameters of relaxation time and endogenous frequency accounted for differences between the beat-deaf cases as well as the control group individuals.

## 1. Introduction

Tapping or clapping to a beat is an easy task for most individuals. This natural perception–action coupling reveals precise temporal synchronization with a variety of familiar and unfamiliar auditory patterns, even in the presence of complex signals that contain temporal fluctuations such as those found commonly in music. Regular clapping to music reveals the temporal constancy that most listeners experience, even though a regular ‘beat’ may not be evident from examination of the acoustic signal [1]. Listeners' ability to track a beat, called beat-tracking, is modelled with a theoretical concept of *pulse*: a perceived regular beat that is generated by an internal oscillation, thought to form the foundation of internal timekeeping mechanisms [2–4]. Explanations of the mechanism underlying beat-based entrainment posit that listeners make adjustments, called adaptations, to an internal (endogenous) oscillator that changes its phase and period in response to the elapsed intervals between stimulus onsets.

Beat-based entrainment abilities are thought to be universal, spontaneous and present from an early age [5]; these abilities underlie group synchronization behaviours such as marching or rowing, and in performing arts such as music or dance, behaviours which are absent from the natural repertoire of non-human species [6]. The human ability to perceive a regular beat in a complex auditory stimulus like music offers natural evolutionary advantages of coordinating one's behaviour with a group through mutual entrainment, sometimes with an external stimulus provided by a coxswain in rowing or a conductor in musical ensembles, and sometime with no external stimulus beyond other group members [7]. Not all humans, however, can track the beat. Beat deafness often refers to self-identified difficulties in tracking or moving to a beat in an external stimulus like music or a metronome. Although prevalence estimates of beat deafness are not yet established, a few clinical cases have been documented; Phillips-Silver *et al.* [8] reported a case, Mathieu, who presented himself as having difficulty following a beat. His performance on a range of perception and production tasks demonstrated rhythm-specific deficits in certain auditory discrimination tasks and deficits in synchronization tasks as he moved his body to a musical beat. Beat-tracking measurements from a large sample of individuals indicate a broad range of temporal entrainment that suggests occasional difficulties in sensorimotor integration [9]. Deficits in beat-tracking are of special interest for several reasons: they shed light on theories of temporal adaptation and timekeeping mechanisms, and they provide tests of neurological underpinnings shared by perceptual and motor tasks that require temporal adaptation.

Beat deafness may reflect a basic failure to anticipate or predict the beat; beat-tracking behaviours typically demonstrate an anticipatory response in advance of the stimulus, yielding negative asynchronies. Alternatively, beat deafness may be more reactive, as reported in the cockatoo Snowball's beat-tracking with head movements [10], or may show tracking tendencies at a preferred endogenous rate, as exhibited in a trained sea lion's head movements [11] and those of budgerigars [12]. Humans' anticipatory tendencies are considered so far to be unique, even if beat-tracking is not [13]. We examined the possibility that beat-deaf individuals anticipate less, or exhibit stronger influences of preferred endogenous rates, by comparing their behaviour with those of a control group in terms of tapping synchronization.

Difficulties in maintaining synchrony with a regular beat may also arise from failures in error correction mechanisms. Error correction refers to a resetting mechanism assumed to adjust the internal reference interval with which the participant is tapping, on the basis of feedback about the asynchrony generated by the previous tap relative to the stimulus onset. In the absence of an error correction mechanism, synchronization error will accumulate over time [14]. The fact that most listeners tap with anticipatory error and yet do not drift away from the stimulus timing provides support for error correction processes. Two forms of error correction have been identified from tapping tasks: phase correction and period correction. Studies of error correction typically use a perturbation task, which introduces unpredictable changes in a tone's period or phase during the presentation of a temporally regular series of tones [14–16]. The experimental goal of perturbations is to elicit a corresponding change in an internal oscillator's phase or period. Tappers' adaptation to perturbations is measured in their relaxation time: the time needed for tapping to return to synchrony with the stimulus following the perturbation. Tappers' relaxation time following phase perturbations (sudden changes to a single tone's onset) is usually quick, with a characteristic pattern of an overshoot (e.g. a delayed tap onset) followed by an undershoot (e.g. a premature tap onset), and is thought to reflect an automatic adjustment to the phase of an internal oscillator, unconstrained by perceptual detection thresholds [16–18]. By contrast, relaxation time following period perturbations (stable changes to the tone period in a series of tones) is usually gradual, largely independent of phase-locking responses and thought to reflect an effortful adjustment of an internal oscillator's period (cf. [19]). We compared adaptation to stimulus phase and period perturbations to test the hypothesis that beat deafness exhibits error correction deficits: specifically, adaptation deficits in response to more effortful period perturbations, with intact automatic adaptation to phase perturbations.

Maintaining synchrony with a changing beat also depends on whether the stimulus period is increasing (slowing down) or decreasing (speeding up). Loehr *et al.* [20] modelled skilled pianists' ability to synchronize with a metronomic (regular) beat that contained a linear period change. In contrast to linear timekeeper models that predict equivalent adaptation to stimulus period changes that slow down or speed up, a nonlinear dynamical systems model [21] predicts faster adaptation to increasing stimulus period than to decreasing period [20], due to the nonlinearity of the oscillator's period adaptation function; the asymmetry increases as the size of the tempo change increases. Indeed, the pianists were better able to adapt to increased stimulus periods than to decreased stimulus periods. If beat deafness reflects deficits in an internal oscillator's period adaptation, then beat-deaf cases may fail to display the predicted asymmetry compared with control participants, a second hypothesis we test here.

Listeners' abilities to adapt their synchronization in response to auditory perturbations have been modelled dynamically in terms of adjustments to the phase and period of an internal oscillator. Large *et al.* [16] modelled six tappers' adaptation to a perturbation task that presented unpredictable phase and period perturbations; a damped harmonic oscillator with primary parameters of relaxation time and intrinsic (endogenous) period was applied to the relative phase measures of temporal adaptation. The harmonic oscillator model fits indicated faster relaxation times in response to phase perturbations than to period perturbations, consistent with the behavioural findings described earlier [16]. If beat-deafness entails adaptation failures in relaxation time or intrinsic oscillator period, then the two parameters may display smaller values for beat-deaf cases relative to control participants, a third hypothesis we tested here by fitting a damped harmonic oscillator model to the tapping responses.

We compared the performance of Mathieu [8] with age- and education-matched controls, using a perturbation tapping task [16]. In addition, we introduce a second beat-deaf case, Marjorie, who identified herself as having difficulty tracking a beat. First, participants tapped regularly at a comfortable rate in the absence of any stimulus, to yield a measure of their spontaneous motor tempo. This task allowed us to document any deficits in the absence of external auditory stimuli, and provided a measure of an endogenous preferred rate, which may dominate adaptation responses in the absence of error correction abilities, as suggested by beat-tracking in non-human species [12]. Second, participants synchronized with a regular metronome; this task allowed us to determine how error correction mechanisms operated in the presence of a metronomically regular beat. Finally, we introduced unpredictable (random) perturbations that changed the stimulus phase or period of a regular auditory stimulus in the perturbation task. The rate at which the control participants and beat-deaf cases adapted to the stimulus perturbations allowed us to test the three hypotheses: (i) whether the beat-deaf individuals exhibited faster adaptation to phase perturbations than to period perturbations as expected for the control group; (ii) whether the beat-deaf individuals exhibited the expected faster adaptation to increased (slowed) than to decreased (speeded) stimulus period perturbations and (iii) whether endogenous frequency or relaxation parameters of a simple harmonic oscillator model could account for deficits in beat-tracking.

## 2. Material and methods

### (a) Participants

Two ‘beat-deaf’ individuals responded to a recruitment advertisement for people who had difficulty keeping a beat in music, for example, when dancing or clapping in time at a concert. Mathieu reported 3 years of informal vocal training and 1 year of guitar training (see Phillips-Silver *et al.* [8] for further details). Marjorie reported no formal musical training beyond group instruction in primary school. Mathieu and Marjorie were screened for amusia with the Montreal Battery of Amusia [22]. Their scores on all tests, including rhythm discrimination, were in the normal range; the exception was the meter test (Mathieu: 67% accuracy; Marjorie: 43% accuracy, controls' mean: 86% accuracy, based on norms of [22]), in which they had to determine whether the pattern of strong and weak beats in short unfamiliar musical pieces corresponded to a march (binary accent pattern) or a waltz (ternary accent pattern). Thirty-two control participants (adult males) were recruited; they were similar to the beat-deaf individuals in age and education (table 1). Although the control participants were not selected for musical training, neither beat-deaf individual differed significantly from the control group in musical training. To confirm that years of musical training did not influence the comparisons, the analyses were repeated for the control participants with less than 3 years of musical training (*n* = 26, mean = 0.9 years); each of the comparisons in table 1 did not change in significance, indicating that musical training alone did not account for the differences. This reduced control group showed the same pattern of results in the perturbation task as reported below for the entire (*n* = 32) control group.

**Table 1.** Means and standard deviations for demographic information, spontaneous motor tempo and auditory metronome tapping tasks for control group and beat-deaf individuals. Bold: Crawford's *t*, *p* < 0.05.

variable	controls	Mathieu	Marjorie
age (years)	23.3 (2.59)	24	**30**
education (primary/sec/postsec, years)	14.4 (1.96)	15	16
individual musical training (years)	2.12 (3.04)	1	0
spontaneous motor tempo:			
mean ITI (ms)	749.3 (225)	569.5	852.9
CV of ITI (s.d./ mean)	0.0476 (0.0181)	0.0718	0.0730
regular metronome:			
mean ITI (ms)	499.9 (1.2)	500.0	**504.9**
CV of ITI (s.d./mean)	0.036 (0.008)	**0.059**	**0.118**
mean asynchrony (ms)	−20.0 (27.2)	−28.0	−**81.1**

### (b) Stimuli

Participants heard the auditory stimuli over AKG K271 studio headphones, and their tapping responses were recorded on a silent Roland RD700 electronic keyboard. The spontaneous motor tempo task required participants to tap at a regular pace (in the absence of any auditory stimulus) for 30 s. The regular auditory metronome task presented a stimulus composed of 60 metronome clicks in a drum timbre of 50 ms duration, separated by interonset intervals (IOIs) of 500 ms, lasting 30 s in total over one trial. The auditory perturbation task presented six tone sequences used in Large *et al.* [16], consisting of 306–331 tone onsets each, with a fundamental frequency of 238 Hz and a woodblock timbre. Each trial first presented a regular 500 ms beat period for 12–15 tone onsets to allow participants to settle into a steady-state tempo. Six phase and six period perturbations were then introduced. A phase perturbation was defined as a change in a single IOI followed by a return to the 500 ms IOI. A period perturbation was defined as a single uniform change in successive IOIs for 13–20 onsets, followed by a return to the 500 ms IOI. Each perturbation (phase or period) was an increase or decrease of 3% (±15 ms), 8% (±40 ms) or 15% (±75 ms) from the 500 ms IOI. Each perturbation episode was succeeded by a return to the baseline 500 ms IOI for 13–20 onsets. The perturbation task contained six unique trials, each containing six phase and six period perturbations as well as returns to baseline between perturbations. Duration and order of the perturbations within each trial were pseudo-randomly generated, and the order of the trials was counterbalanced between participants.

### (c) Procedure

Each participant completed the spontaneous motor tempo tapping task, the regular metronome task and the perturbation task in that order, using the index finger of their dominant hand. Participants' spontaneous tapping rate was measured by asking participants to tap at a regular and comfortable pace on the silent piano keyboard for 30 s. Participants then synchronized their taps with the regular metronome stimulus. Then participants completed the perturbation task in which they were instructed to synchronize their taps with an auditory sequence in which the time between beats might change. Participants completed a questionnaire about their musical background and received a small compensation for their participation.

### (d) Statistics

The spontaneous motor tapping task was evaluated in terms of mean and coefficient of variance (CV = s.d./mean) of IOIs. The regular auditory metronome task was evaluated in terms of both IOI and asynchrony measures (tap onset *T_n_* minus stimulus onset *S_n_*), after the first 12 taps were excluded; negative values indicated the tap preceded the stimulus. The auditory perturbation task was evaluated, following Large *et al.* [16], by converting asynchrony measures into relative phase as [(*T_n_* − *S_n_*)/(*S_n_*_+__1_ − *S_n_*)](mod_−0.5,0.5_ 1). Relative phase values ranged from −0.5 to +0.5; negative values indicated the tap preceded the stimulus and positive values indicated the tap followed the stimulus. Values were computed for the 12 beats following each perturbation and were averaged for each participant across the trials within each perturbation type (phase versus period), amount (3, 8 and 15%) and direction (decreased versus increased IOI), as well as within the baseline portion of each trial (12–20 successive beats that contained no perturbation). Participants occasionally omitted a tap (control group: 0.2% of all stimulus taps; Mathieu: 0.5%; Marjorie: 3.8%), in which case the corresponding stimulus beat was omitted and the following stimulus tap was included in the analysis. The baseline values were subtracted from the perturbation values within each condition to evaluate whether performance returned to baseline after each perturbation [16]; an adjusted relative phase of 0 indicates a return to baseline synchronization performance for that individual. Analyses of variance (ANOVA) were conducted on mean relative phase values separately for control participants (treating participant as the subject variable) and for each of the beat-deaf cases (treating trial as the subject variable), with within-subject factors of perturbation type (phase or period), amount (3, 8 and 15%), direction (increasing/decreasing IOI) and serial position after perturbation (1–12). Crawford's modified *t*-tests, with d.f. = 31, one-tailed, were used to compare the beat-deaf cases with norms derived from the control sample [23,24], a conservative test which reduced the likelihood of a type-1 error rate by providing a point estimate of the abnormality of scores from a control sample while taking into account the non-normal distribution of small samples.

## 3. Results

### (a) Spontaneous motor tempo and metronome tasks

Control participants and beat-deaf individuals did not differ significantly in the means or coefficients of variance in their spontaneous motor tempo (table 1). Both Mathieu's and Marjorie's performance on the spontaneous motor tempo task fell within the range of the control group. The 30 s metronome tapping task yielded similar patterns for mean inter-tap intervals between the control participants and the beat-deaf cases (table 1). Synchronization tapping with the metronome, measured by asynchrony of tap onsets minus stimulus onsets, was less well synchronized in the beat-deaf cases but still anticipatory; Mathieu, Marjorie and control participants all showed negative asynchronies consistent with anticipatory behaviour reported in other tapping tasks [25].

Both beat-deaf cases differed from control participants in their temporal variability in the metronome task; as shown in table 1, control participants showed small variability (mean CV = 0.04) and displayed significantly smaller CV values than both Mathieu, *t* = 2.89, *p* < 0.01, and Marjorie, *t* = 10.25*, p* < 0.01. Thus, both beat-deaf cases were less precise than the control group, and one case (Marjorie) was also less synchronous on average in tapping with a regular metronome.

### (b) Perturbation task

Comparisons of synchronization during the baseline portion of each trial (in the absence of any perturbations) confirmed that the control participants were able to maintain synchronization, while the beat-deaf cases displayed significantly greater variability (Mathieu: *t* = 4.26, *p* < 0.001; Marjorie: *t* = 4.31, *p* < 0.001). The mean relative phase values during the baseline portion of each trial differed from the control group only for Marjorie (control group = −0.0941; Marjorie = −0.2066, *t* = −2.14, *p* < 0.01). This pattern resembles the same distinctions shown in table 1; both beat-deaf cases are more variable, and only one case differs in mean tendency from the control group. Importantly, this finding verifies that both beat-deaf cases are in fact anticipating the beat, similar to the control group individuals.

**Figure 1.**
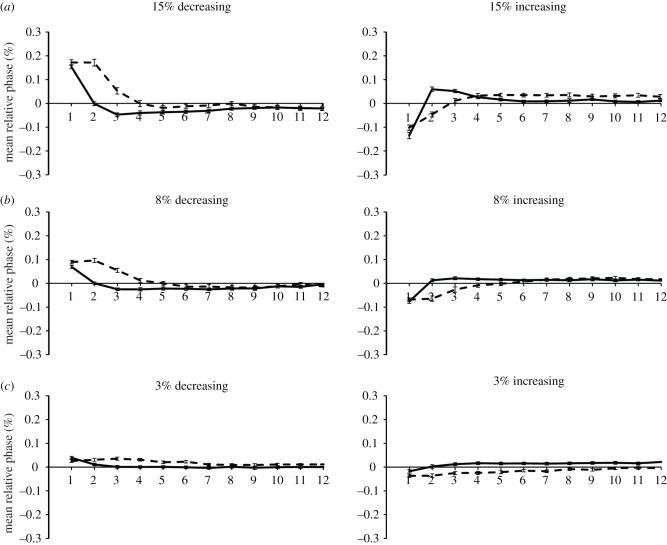
Mean relative phase values for control participants by perturbation type (solid lines represent phase and dashed lines, period), direction, amount and sequence position following perturbation.

The mean relative phase values for control participants are shown in figure 1 by condition. The control participants' relative phase values moved away from 0 and relaxed back to 0 usually within four beats for both perturbation types and directions. Period perturbations relaxed to 0 smoothly and gradually, whereas phase perturbations were typically over-corrected, resulting in a change of sign in relative phase before returning to baseline. Significant main effects of perturbation type, amount and serial position (*p* < 0.05) were observed, as were additional interactions; we focus on those here with respect to the two primary hypotheses.

First, the overcorrecting pattern expected for phase perturbations more than period perturbations was confirmed for the control participants in the significant interaction between perturbation type and position (*F*_11,341_ = 2.81, *p* < 0.01), the interaction between perturbation type and amount (*F*_2,62_ = 14.28, *p* < 0.001) and the three-way interaction between these variables (*F*_22,682_ = 1.68, *p* < 0.05). Second, the predicted tendency to adapt faster to decreased than to increased stimulus periods was confirmed in the interaction of direction with position (*F*_11,341_ = 178.65, *p* < 0.001) and in the three-way interaction of direction, position and amount (*F*_22,682_ = 73.58, *p* < 0.001). Finally, there was a four-way interaction between perturbation type, amount, direction and position (*F*_22,682_ = 24.64, *p* < 0.001); ANOVAs repeated within each perturbation amount (3, 8 and 15%) indicated that the same significant interactions were present at 8 and 15% perturbations, whereas perturbation type by position effects were absent for 3% perturbations, indicating they were too small to generate much response.

**Figure 2.**
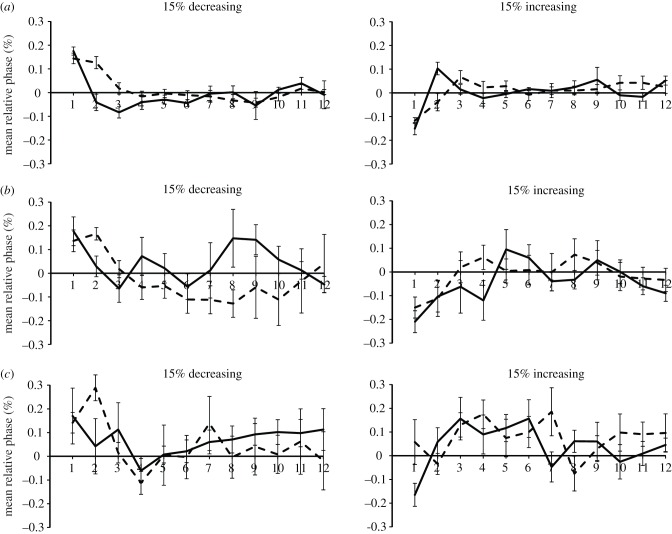
Mean relative phase values for 15% stimulus change (decreasing and increasing IOIs) for control participant (*a*), Mathieu (*b*) and Marjorie (*c*). Solid lines represent phase perturbation and dashed lines, period perturbation.

Mean relative phase values for Mathieu and Marjorie are shown in figure 2, along with a representative control group participant, for the 15% perturbation conditions. Both beat-deaf individuals showed a failure to return to baseline following phase or period perturbations. Mathieu's mean relative phase values for phase and period perturbations are very similar; he did not respond differentially to perturbation amount (3, 8 or 15%) and, critically, did not show the expected interactions between perturbation type and position consistent with the first hypothesis: the predicted faster recovery with over-correction patterns typical of phase perturbations. Mathieu's data exhibit significant interactions between direction and position (*F*_11,55_ = 5.36, *p* < 0.001) and a three-way interaction between direction, perturbation and amount (*F*_2,10_ = 7.00, *p* < 0.05); ANOVAs repeated within each perturbation amount indicated a differential response to direction only for 15% perturbations. Thus, the hypothesis that phase perturbation responses would be intact was not supported for Mathieu, whereas the hypothesis that relaxation times would be faster for increased stimulus periods received only partial support from Mathieu's relaxation responses to the largest (15%) stimulus perturbations.

Marjorie showed similar failures to return to baseline following both phase and period perturbations, as shown in figure 2*c*; her mean relative phase values did not change across perturbation types or amounts, also failing to support the first hypothesis. She did show differential response to perturbation direction, indicated by a significant direction by position interaction (*F*_11,55_ = 2.13, *p* < 0.05), and by a three-way interaction with amount (*F*_22,110_ = 2.26, *p* < 0.01). ANOVAs repeated within each perturbation amount confirmed her faster adaptation to increased than to decreased stimulus IOIs, consistent with the second hypothesis. Thus, both beat-deaf cases failed to adapt differentially to phase perturbations versus period perturbations, and only Marjorie responded differentially to increased and decreased perturbations across perturbation amounts, like the control group. These findings suggest both similarities and differences in the types of disruption exhibited in the beat-deaf cases, which we test further with computational models.

### (c) Model fits

To further investigate the underlying mechanisms differentiating the control participants and beat-deaf cases, the relaxation curves were fit with a damped harmonic oscillator model [16]. If the two beat-deaf cases exhibit different types of disruption, we expect that the model's parameter fits may exhibit different values relative to each other and to the control group. Equation (3.1) shows the oscillator's predicted response in relative phase to a stimulus onset, in which *A* is the amplitude of the perturbation; *b*, relaxation time; *f*, internal frequency; *n*, tap position; and *θ*, position within cycle
3.1
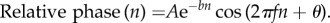

Each control participant's relative phase data for each condition was fit by the model, following the initial parameter settings of *A* = perturbation amounts (0.03, 0.08 and 0.15) and *θ* = 0. The parameters *b* (constrained to greater than 0 with initial value = 1.0) and *f* (constrained to greater than or equal to 0 with initial value = 0.15) were fit using a Levenberg–Marquandt nonlinear least squares procedure, following Large *et al.* [16], to address the following hypotheses: (i) the relaxation time parameter 1/*b* was expected to differ for phase and period perturbations, such that larger values of *b* should capture faster relaxation for phase than for period perturbations; and (ii) values of *b* should be larger for increased stimulus IOIs than for decreased stimulus IOIs, following Loehr *et al.* [20]. With respect to the internal oscillator frequency, *f*, we hypothesize that (iii) values of *f* should be larger in response to increased than to decreased stimulus IOIs.

**Figure 3.**
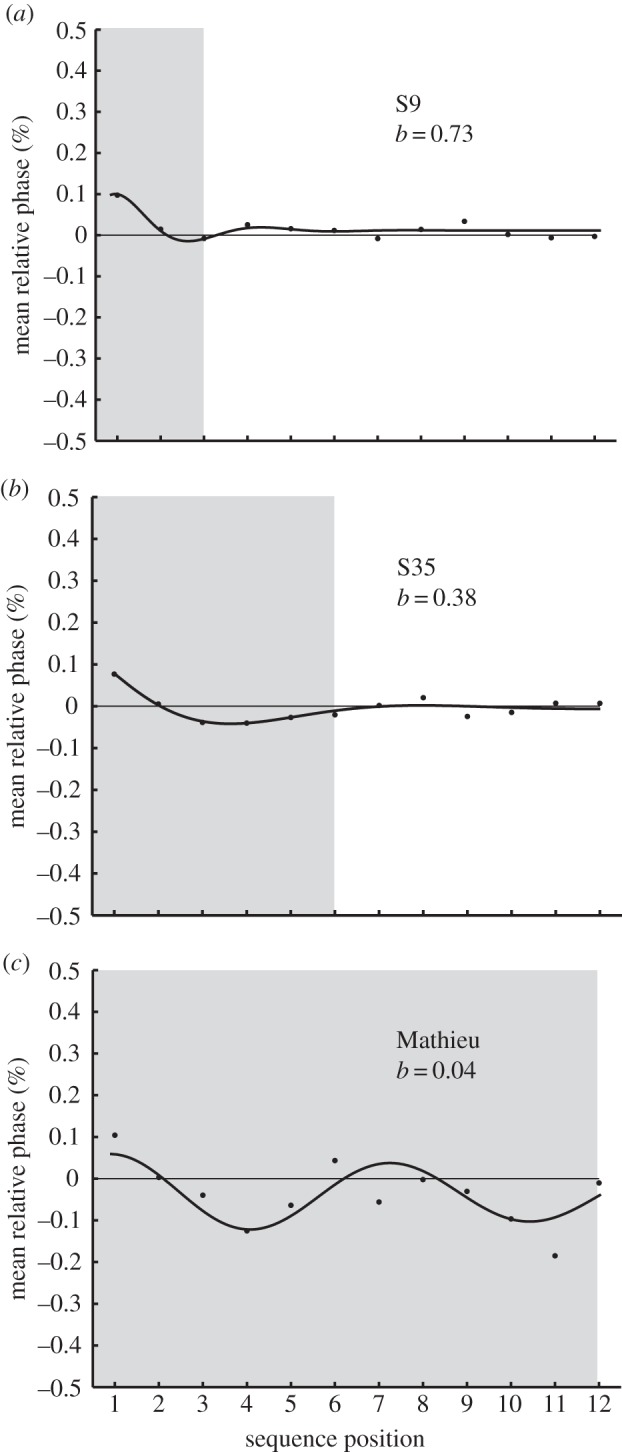
Model fits (line) to data (dots) from sample trials (8% phase perturbation) of control participants and Mathieu. Shaded region demonstrates relaxation parameter *b* for fast (*a*), moderate (*b*) and slow (*c*) adaptation.

The model's goodness of fit was measured with variance accounted for (VAF) and the Akaike Information Criterion (AIC; [26]), an ordinal-scale measure in which small values indicate better fits. The model's overall goodness of fit was significant for each of the control participants (mean VAF = 83%; *p* < 0.01; mean AIC = −69.04) and accounted for more variance in the data for the 8% (mean VAF = 87%) and 15% (mean VAF = 93%) perturbations (*F*_2,62_ = 87.51, *p* < 0.001) than for the 3% perturbations (mean VAF = 69%). The majority (91%) of individual fits that did not reach significance were from the 3% perturbation condition, which generated relatively little disruption in tappers' responses (figure 1*c*); further model comparisons were computed only for the 8 and 15% perturbation conditions. Examples of model fits to individuals with high and low *b* values representing fast and slow adaptation, respectively, are shown in figure 3*a*,*b*. There was a significant main effect of perturbation type (*F*_1,31_ = 9.31, *p* < 0.01), with faster relaxation time (larger *b*) for phase shifts (mean *b* = 0.8321) than for period shifts (mean *b* = 0.6334), as predicted. Values of *b* were larger (faster relaxation) for increased stimulus IOI than for decreased stimulus IOI (*F*_1,31_ = 6.71, *p* < 0.05), also as predicted [20]. Thus, these results extend the damped harmonic oscillator model [16] to fits of a larger group of participants, and confirm the hypotheses for the control group.

**Figure 4.**
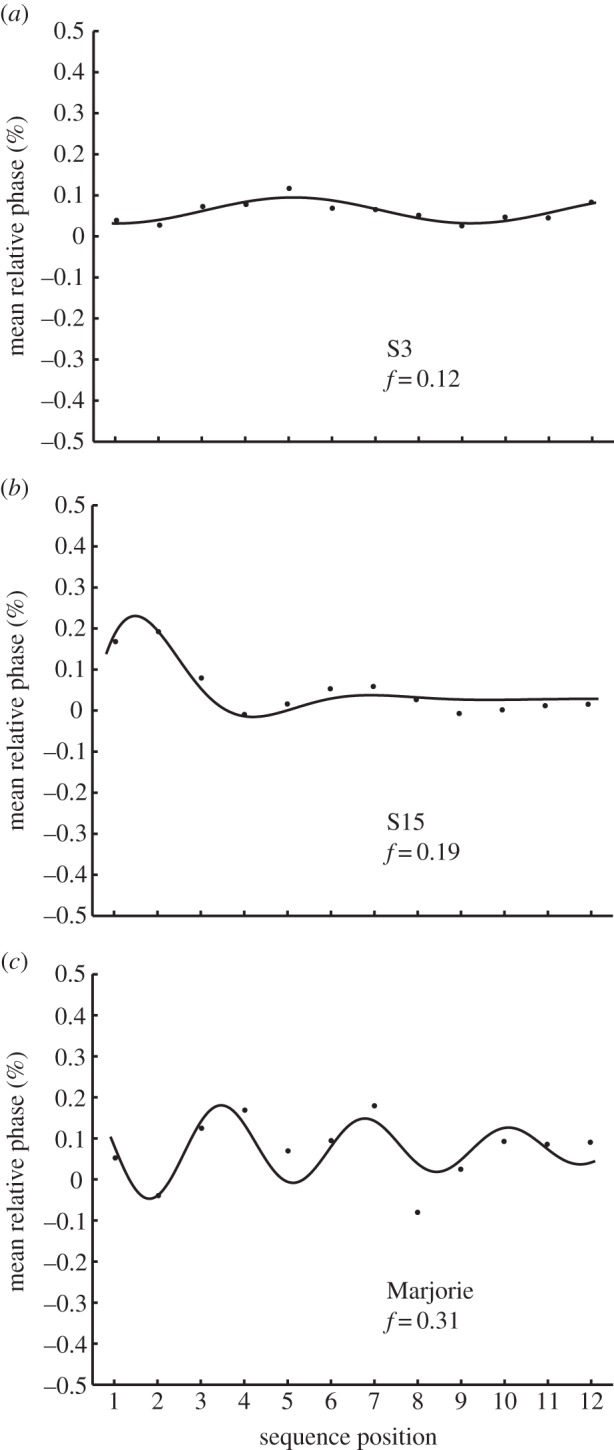
Model fits (line) to data (dots) from sample trials (15% period perturbation) of control subjects and Marjorie. Internal oscillation frequency values *f* demonstrate slow (*a*), moderate (*b*) and fast (*c*) frequencies.

Examples of model fits to individuals with low and high *f* parameter values representing slow and moderate oscillator frequencies, respectively, are shown in figure 4*a*,*b*. Control participants' *f* values were larger for period perturbations than for phase perturbations (*F*_1,31_ = 21.61, *p* < 0.01). The oscillatory fluctuations in responses were greater for increased stimulus IOI than for decreased stimulus IOI, as expected (*F*_1,31_ = 9.60, *p* < 0.01). The *b* and *f* parameter values for each individual, averaged across conditions, were not correlated across control participants (*r*_30_ = −0.19, *p* > 0.10), indicating that the two parameters accounted for different aspects of temporal adaptation. Neither the amplitude (*A*) nor the cycle position, *θ*, showed the same differences across conditions for the control group or the beat-deaf cases.

Overall, the harmonic oscillator model provided significant fits to the same perturbation conditions in the beat-deaf cases (Mathieu's fits: mean VAF = 70%, AIC = −40.0; Marjorie's VAF = 69%, AIC = −41.7; *p* < 0.01); the model did not fit as well as to the control group individuals. The fitted parameter for relaxation time, *b*, shown for one of Mathieu's trials in figure 3*c*, indicates slower recovery following perturbations. The model parameter values were compared between the beat-deaf and control individuals for the 15 and 8% perturbation conditions (the conditions that generated adaptation responses in the control group). Model fits indicated significantly smaller *b* values for Mathieu (mean *b* = 0.2869, *t* = −1.81, *p* < 0.05) and Marjorie (mean *b* = 0.2707, *t* = −1.871, *p* < 0.05) than the fits to the control group individuals, indicating that the harmonic oscillator captured slower adaptation by the beat-deaf individuals in response to perturbations.

The model's fit demonstrating a large internal oscillation frequency, *f,* is shown for one of Marjorie's trials in figure 4*c*; it indicates a fast oscillatory response following perturbations. Comparisons of the *f* parameter values between the beat-deaf and control individuals indicated significantly larger values for Marjorie than those of the control group individuals (mean *f* = 0.4683, *t* = 1.78, *p* < 0.05), whereas Mathieu's values did not differ significantly from the control group. Thus, the model predicted increased oscillatory behaviour relative to control participants following all perturbation types in Marjorie's case.

Are different parameter values from the model's fits to the beat-deaf cases due to specific adaptation mechanisms or simply to general increases in noise? The smaller relaxation values, *b*, for the beat-deaf cases indicate a decreased (shallow) slope that may reflect noise in their responses. To test the possibility that increased variance in the relative phase values alone may account for changes in model parameter values, we contrast the model's fit to original data with that of simulated data: the simulated relative phase values for each individual and condition were drawn from a uniform distribution with mean of 0 and variance equal to the obtained variance for that individual/condition. If the harmonic oscillator model generated fits to the simulated data with parameter values that showed the same differences across observed data, then we cannot rule out the possibility that noise alone accounted for the fits. The model fits to simulated data were worse for all individuals (control group: mean VAF = 48%; Mathieu: mean VAF = 49%; Marjorie: mean VAF = 42%; *p*'s < 0.05) than to the observed data. Furthermore, the values of *b* for simulated data did not differ for perturbation type, amount, or direction, as was seen in the model's fit to observed data. Thus, these findings argue against the possibility that the model changes in temporal relaxation parameter values were simply due to increased noise in the adaptation responses.

Finally, the damped harmonic oscillator model was compared with other models of perturbation tasks. Large *et al.* [16] weighted the first four taps after each perturbation more heavily than the remaining data; weighting of the first four taps did not improve the model fit significantly beyond the unweighted model reported here. The damped harmonic oscillator model was also compared with the exponential decay model of Pfordresher & Kulpa [27] that contained additive (linear) slope and intercept (asymptote) parameters; this model provided a significant fit to data of only 13 of 32 control participants (mean VAF = 63%, *p* > 0.05; mean AIC = −61.3) and to neither of the beat-deaf individuals (Mathieu's fits: mean VAF = 43%, *p* > 0.05; mean AIC = −38.9; Marjorie's fits: mean VAF = 38%, *p* > 0.05; mean AIC = −3.0); nor did the model explain the differences across perturbation conditions.

## 4. Discussion

We compared two beat-deaf individuals with a large group of control participants in their ability to coordinate their tapping behaviour with an auditory stimulus. Using a perturbation task designed to disrupt the internal state of an oscillatory dynamical system, we measured the speed with which they adapted in response to the changing beat. The synchronization behaviour was modelled with a damped harmonic oscillator, whose parameters of relaxation time and endogenous oscillator period accounted for differences between the control group and the two beat-deaf individuals. Together, the empirical and computational findings illustrate that the hypothesis of intrinsic dynamical oscillations provides a useful model for both accurate coordination and for deficits in beat-tracking.

Listeners' adaptation to temporal fluctuations in the sequences with which they coordinated their tapping differed with the type of perturbation. Evidence from a control group replicated findings that people quickly adjust to phase perturbations, but adjustment to period perturbations is slower [16,18]. We documented two beat-deaf individuals' adaptation abilities in response to phase and period perturbations, suggesting a broader deficit than one specific to phase or period adaptation. Importantly, their responses, like those of the control group, were anticipatory, preceding the stimulus beat. Also important is the fact that the beat-deaf cases showed greater variance than control participants in the presence of a regular auditory metronome, suggesting that prediction tendencies alone cannot account for the specific deficit.

Listeners' adaptation to temporal perturbations also differed with the size or amount of perturbation; listeners exhibited greater adaptation to larger perturbations (15% of the base IOI) and little adaptation to the smallest (3%) perturbations. Large *et al*. [16] reported similar findings for six (control) participants, for the same range of perturbations. Other studies, which focused on expert tappers chosen for their ability to synchronize with low variability [28], have reported greater adaptation in response to smaller (2%) perturbations than the ones presented in this study.

A nonlinear dynamical approach to temporal coordination assumes that an external rhythmic signal evokes intrinsic neural oscillations that entrain to the periodicities in the rhythmic sequence [3,15]. Both the phase and the period of an internal oscillator must be adjusted in order to maintain coordination in the face of tempo changes in an external sequence [16,20]. Phase and period adaptation mechanisms are assumed to be updated for each stimulus event, based on synchronization error computed between the previous stimulus event and response, termed error correction. A failure to adjust either the phase or period adaptation mechanisms would essentially impede an individual's ability to correct their behaviour based on error signals. Both beat-deaf individuals exhibited error correction failures to both period and phase perturbations; these characteristics should negatively affect the production of any timed synchronization behaviour with an external stimulus. This interpretation is consistent with the observed intact mean tempo in the beat-deaf cases' spontaneous motor tempo task (which does not require correction to an external stimulus) and larger variance in the regular metronome task (which does require correction to an external stimulus).

Both beat-deaf and control individuals adapted faster to sequences whose rate slowed down (increased IOI) than one whose rate speeded up (decreased IOI). Loehr *et al*. [20] proposed a temporally discrete oscillator model that predicted synchronization with a changing metronome with two independent parameters: phase coupling strength and period adaptation strength. The nonlinear model's prediction that temporal adaptation is faster for sequences that slow down arises from the nonlinearity of the period adaptation function; the predicted asymmetry increases as the size of the tempo change increases [20]. We replicated that asymmetry in the context of the perturbation task; listeners' relaxation time was faster for increased stimulus IOIs (slowing) than for decreased IOIs, for both period and phase perturbations. The beat-deaf individuals exhibited the same asymmetry, primarily in response to large perturbations. Their findings suggest that some period adaptation is possible, although with significantly less sensitivity than in the control group. Future work may test whether phase adaptation and period adaptation parameters show equivalent deficits in a larger sample of beat-deaf cases.

The two beat-deaf individuals showed important distinctions in their beat-tracking abilities. Whereas Mathieu's failures of adaptation in response to perturbations indicated abnormal relaxation time, Marjorie exhibited deficits both in relaxation time and in intrinsic oscillator frequency. Her adaptation patterns were characterized by high-frequency oscillations that lasted several seconds, distinct from the adaptation functions of control participants and Mathieu. Measures of intrinsic frequency have been shown to influence synchronization behaviours in both humans [29] and animals [11,12]. The different deficits shown by beat-deaf cases also provide independent support for the harmonic oscillator model; neither parameter alone could account for the deficits, suggesting that beat deafness is a spectrum of temporal coordination disorder. Consistent with this view, Mathieu is able to identify a regular beat in simple auditory stimuli but displayed difficulty in tracking the beat in more complex musical stimuli [8]. Also consistent with this view, a range of phase adaptation and period adaptation parameters have been reported for skilled musicians' abilities to track a changing metronome, and a broad range of temporal adaptation abilities in tapping behaviours have been reported for a large sample of normal controls [9]. Thus, the control group and the beat-deaf individuals can be viewed as spanning a continuum of error correction mechanisms, and specifically, differences in internal period coupling mechanisms, a hypothesis that may be tested in future cases of beat deafness.

In sum, tapping to the beat is a natural sensorimotor coupling activity for most individuals. Rare cases of beat deafness show deficits of temporal adaptation that provide opportunities to test computational models of underlying neural mechanisms of timekeeping. A damped harmonic oscillator model captured the differences between two beat-deaf cases and a control group in their ability to adapt to unpredictable temporal perturbations; the model accounted for the major differences and confirmed the entrainment failures reported previously [8] for one of the beat-deaf individuals, Mathieu. The fact that both beat-deaf cases exhibited normal spontaneous motor tempi and exhibited increased variance with a regular auditory metronome supports a deficit specific to error correction in perception–action coupling. How these individuals adapt to other temporally unpredictable events, including conversational speech and joint action tasks, is a question for further study.

## Supplementary Material

Palmer et al. 2014 Simulation

## Supplementary Material

Palmer et al. 2014 Model
